# Electric-field engineering of band alignment and photovoltaic properties in a BSe/HfSO van der Waals heterostructure

**DOI:** 10.1039/d6na00716c

**Published:** 2026-11-03

**Authors:** Tran T. Nhan, Pham T. Truong, Le T. Ngoc Tu, Huynh V. Phuc, Chuong V. Nguyen, Ngoc-Trung Nguyen, Trang Minh Pham, Thi Kim Cuong Phu, Tam Duy Nguyen, Yoshiyuki Kawazoe, Thi Viet Bac Phung

**Affiliations:** a Faculty of General Education, Hanoi University of Industry 298 Cau Dien Hanoi 100000 Vietnam tran.nhan@haui.edu.vn; b Division of Physics, School of Education, Dong Thap University Cao Lanh 870000 Vietnam; c Faculty of Mechanical Engineering, Le Quy Don Technical University Hanoi 100000 Vietnam; d Center for Environmental Intelligence, VinUniversity Hanoi 100000 Vietnam; e School of Chemistry, Monash University Melbourne Victoria 3800 Australia; f New Industry Creation Hatchery Center (NICHe), Tohoku University Sendai 980-8579 Japan; g Department of Centre for Inter Disciplinary Research, SRM University-AP Amaravati 522240 Andhra Pradesh India; h College of Engineering and Computer Science, VinUniversity Hanoi 100000 Vietnam bac.ptv@vinuni.edu.vn

## Abstract

van der Waals (vdW) heterostructures have emerged as an effective platform for tailoring the electronic and optical properties of two-dimensional materials through interlayer coupling. In this work, the structural, electronic, mechanical, and optical properties of a BSe/HfSO vdW heterostructure are systematically investigated using first-principles calculations. The calculations reveal that the BSe/HfSO heterostructure possesses a native type-II band configuration and a narrower gap than either isolated monolayer, which may facilitate spatial separation of photoexcited electrons and holes. In addition, formation of the heterostructure substantially strengthens its response in the visible spectral range, yielding an estimated power conversion efficiency of 4.27% and indicating its promise for ultrathin photovoltaic devices. More importantly, its electronic properties can be effectively manipulated by an external perpendicular electric field, resulting in continuous band-gap modulation and an electrically induced transition from type-II to type-I band alignment. These findings demonstrate the electronic and optical tunability of the BSe/HfSO vdW heterostructure and highlight its potential for future optoelectronic and photovoltaic applications.

## Abstract 2

van der Waals (vdW) heterostructures have emerged as an effective platform for tailoring the electronic and optical properties of two-dimensional materials through interlayer coupling.

## Graphical abstract

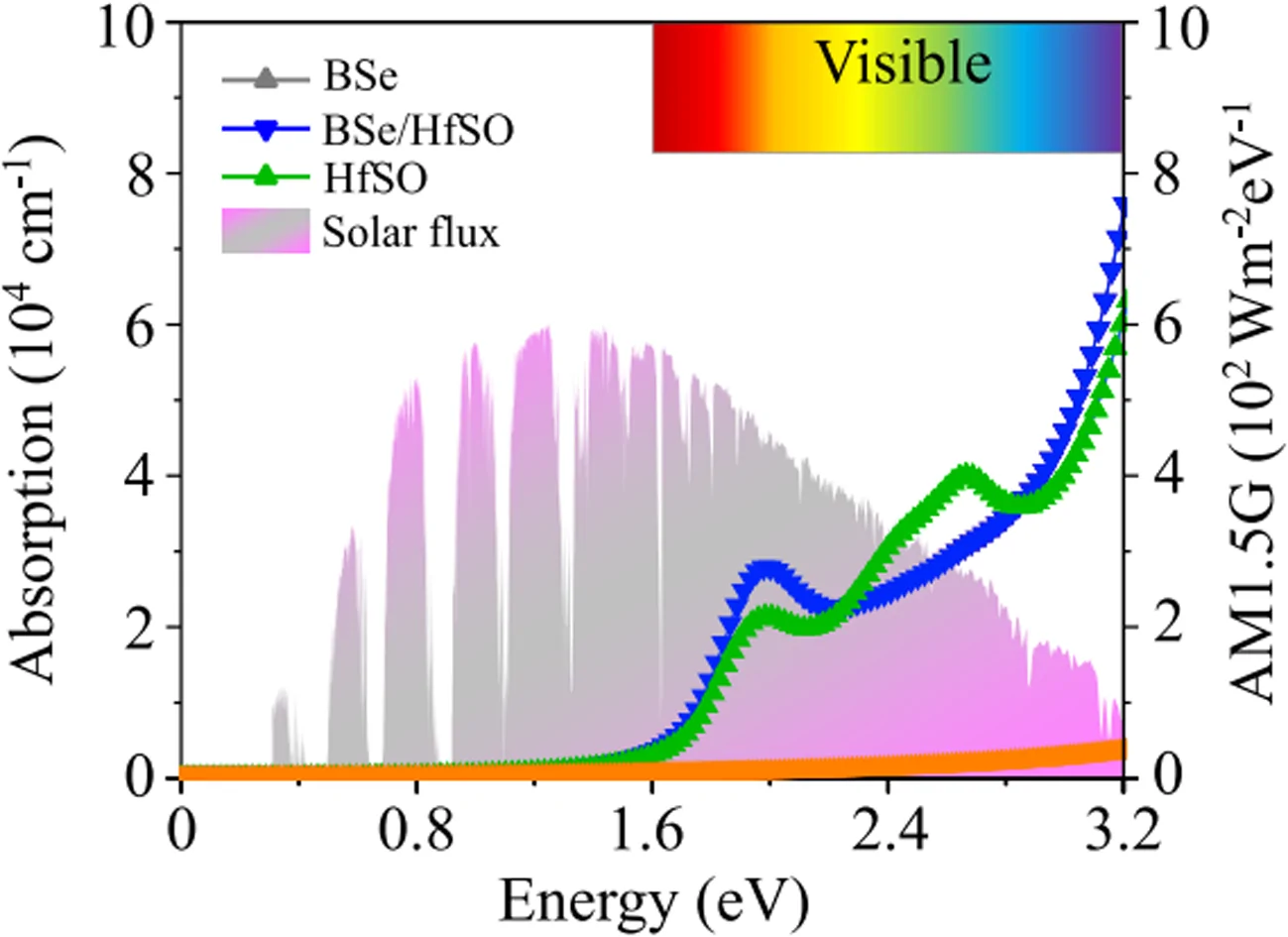

## 1 Introduction

The field of two-dimensional (2D) materials has witnessed remarkable progress since the isolation of graphene,^1^ which marked a paradigm shift in condensed matter physics and materials science.^2^ Graphene, a single atomic layer of carbon arranged in a honeycomb lattice, exhibits extraordinary carrier mobility and thermal conductivity, establishing a foundation for the exploration of other atomically thin systems.^3,4^ Following this breakthrough, a wide variety of 2D materials have been successfully developed, including transition metal dichalcogenides (TMDs)^5,6^ such as MoS_2_ and WS_2_, elemental 2D semiconductors like silicene^7,8^ and phosphorene,^9,10^ hexagonal boron nitride (h-BN).^11^ Collectively, these systems span a broad range of electronic regimes, including metallic, semiconducting, insulating, and superconducting states, thereby opening opportunities in nanoelectronic, optoelectronic, and energy-related technologies.

Despite these significant advances, intrinsic 2D materials still face several fundamental limitations that hinder their standalone application in next-generation devices. For instance, graphene lacks an intrinsic band gap,^12^ which severely restricts its use in digital logic applications.^13^ Many semiconducting 2D materials suffer from relatively low carrier mobility compared to graphene, while others exhibit instability under ambient conditions or limited tunability of their electronic structures.^14,15^ Furthermore, weak light–matter interaction in certain atomically thin layers can limit their optical absorption efficiency, thereby constraining their performance in optoelectronic devices.^16,17^

Several approaches have therefore been developed to modify the intrinsic characteristics of two-dimensional materials and overcome these limitations.^18–25^ One of the most powerful approaches is the construction of van der Waals (vdW) heterostructures,^22,23,26–30^ where different 2D layers are vertically stacked to achieve tailored band alignment and interlayer coupling without the constraint of lattice matching. Other widely used methods include strain engineering,^31,32^ which modulates the electronic band structure through mechanical deformation; chemical functionalization and doping,^18–21^ which introduce additional charge carriers or modify local electronic states; and external field tuning,^33–35^ which enables dynamic control of carrier distribution and excitonic effects. For instance, Wang *et al.*^35^ predicted that the electronic characteristics of the 2D AlN sheet can be tailored by the fluorination and hydrogeneration. Among the various strategies developed to tailor the properties of two-dimensional materials, the construction of vdW heterostructures has emerged as one of the most effective and versatile approaches. Unlike strain engineering, chemical doping, or surface functionalization, which may introduce structural distortions, impurity states, or degradation of carrier mobility, vdW heterostructures preserve the intrinsic atomic structures of the constituent layers while enabling strong interlayer interactions through non-covalent coupling. More importantly, the absence of dangling bonds and the weak vdW interaction at the interface allow atomically thin materials with different lattice constants and crystal symmetries to be integrated without the stringent lattice-matching requirements encountered in conventional semiconductor heterojunctions.

Monolayer boron selenide (BSe) has recently attracted attention as a two-dimensional semiconducting material because of its favorable structural stability and distinctive electronic characteristics.^36^ Previous theoretical studies have revealed that monolayer BSe possesses an indirect band gap of approximately 2.61 eV, thereby making BSe relevant to prospective nanoelectronic and optoelectronic implementations.^36–38^ Its electronic properties and structural compatibility with layered compounds also make BSe a suitable constituent for assembling vdW heterostructures.^39–43^ Several studies have demonstrated that combining BSe with other 2D materials can significantly enhance device performance. For example, Zhu *et al.*^41^ theoretically predicted that the MoSTe/BSe heterostructure exhibits a direct Z-scheme band alignment, making it a promising photocatalyst for solar-driven water splitting. Similarly, Luo *et al.*^39^ reported that BSe/TMDs heterostructures possess improved carrier mobility and enhanced optical absorption compared with their constituent monolayers, highlighting the potential of BSe-based heterostructures for advanced optoelectronic applications.

Meanwhile, Janus two-dimensional materials have attracted tremendous attention as a novel class of atomically thin systems characterized by broken out-of-plane mirror symmetry. This structural asymmetry gives rise to intrinsic dipole moments and highly tunable electronic, optical, piezoelectric, and catalytic properties.^44,45^ To date, a wide variety of Janus 2D materials have been experimentally synthesized or theoretically predicted, including Janus transition-metal dichalcogenides (TMDs),^44,46,47^ Janus MoSH,^45^ and Janus monochalcogenides.^48–50^ Among them, monolayer HfSO, a Janus derivative of transition-metal oxychalcogenides, has emerged as a particularly promising candidate because of its excellent thermodynamic stability and semiconducting characteristics.^51,52^ Previous studies have shown that the band-edge positions of HfSO straddle the redox potentials of water, indicating its potential applicability for photocatalytic overall water splitting.^51^ Nevertheless, the relatively low carrier mobility of HfSO may limit its performance in high-efficiency electronic and optoelectronic devices. To address this limitation, the integration of HfSO with other two-dimensional materials through vdW stacking has recently attracted increasing attention. For example, HfSO-based heterostructures have been predicted to exhibit enhanced charge separation, tunable band alignment, and improved photocatalytic and optoelectronic performance compared with the isolated HfSO monolayer.^53,54^ Nevertheless, these studies on HfSO-based vdW heterostructures have mainly focused on materials interface configurations, while the influence of the intrinsic Janus polarity of HfSO on the electronic properties of both O- and S-terminated interfaces have received comparatively less attention. The intrinsic polarity of Janus HfSO introduces two chemically distinct O- and S-terminated interfaces, whose effects on the electronic properties of BSe/HfSO have not yet been systematically clarified. In particular, the interplay between interface termination, stacking configuration, and interfacial charge redistribution remains insufficiently explored.

In this work, we systematically investigate the structural, electronic, mechanical, and optical properties of the BSe/HfSO van der Waals heterostructure using first-principles calculations. Different stacking configurations with both S- and O-terminated interfaces are examined to identify the most stable geometry. Our results demonstrate that the BSe/HfSO heterostructure exhibits an intrinsic type-II band alignment, enhanced visible-light absorption, and tunable electronic structure, making it a promising candidate for next-generation optoelectronic and photocatalytic applications.

## 2 Computational methods

First-principles calculations were carried out using the Quantum ESPRESSO (version 6.4)^55,56^ based on density functional theory (DFT). Electron-ion interactions were represented within the projector-augmented-wave (PAW) framework^57^ with Vanderbilt pseudopotentials, whereas exchange–correlation effects were incorporated through the generalized-gradient approximation (GGA)^58^ with the Perdew–Burke–Ernzerhof (PBE) functional.^59^ The valence electron configurations explicitly treated in the pseudopotentials are 5p^6^5d^2^6s^2^ for Hf, 3s^2^3p^4^ for S, 2s^2^2p^4^ for O, 2s^2^2p^1^ for B, and 4s^2^4p^4^ for Se. A plane-wave basis with a cutoff energy of 510 eV was adopted for all calculations. Brillouin-zone integrations were performed using a 12 × 12 × 1 *Γ*-centered Monkhorst–Pack mesh. Structural relaxation was continued until the residual Hellmann–Feynman force on every atom was below 0.01 eV × Å^−1^ and the change in total energy between consecutive self-consistent iterations was below 10^−6^ eV. A 30 Å vacuum spacing was introduced normal to the atomic sheets to suppress spurious interactions between periodically repeated slabs. Long-range van der Waals contributions were included through the Grimme DFT-D3 correction during structural relaxation to properly describe the weak interlayer interaction.^60^ To improve the description of the band gap and electronic structure, additional calculations were performed using the screened hybrid Heyd–Scuseria–Ernzerhof (HSE06)^61,62^ functional with mixing parameter of 0.25. Dynamical stability was assessed from phonon dispersions obtained within density-functional perturbation theory (DFPT).^63^ The vibrational properties were computed using a 3 × 3 × 1 supercell to properly capture interatomic force interactions and ensure convergence of the phonon spectra. Thermal stability was subsequently examined through *ab initio* molecular-dynamics simulations (AIMD) in the canonical NVT ensemble controlled by a Nosé–Hoover thermostat. The AIMD trajectories were generated at 300 K for 5 ps using a 1 fs integration interval. The dipole correction was applied along the out-of-plane direction for all considered slab systems. Furthermore, it is emphasized that spin–orbit coupling (SOC) can be important when investigating materials containing relatively heavy elements. In particular, previous first-principles studies of Hf-based 2D materials^64^ have shown that SOC can induce noticeable spin splitting, particularly near the valence-band edges. However, these studies also reported that the inclusion of SOC does not substantially modify the band gap or the fundamental semiconducting nature of these Hf-based materials. Therefore, SOC was not explicitly included in the present calculations.

## 3 Results and discussion

We first examine the structural, electronic, and dynamical characteristics of the isolated HfSO and BSe monolayers before constructing the vdW heterostructure. As depicted in Fig. 1(a), the Janus HfSO monolayer consists of a Hf atomic plane sandwiched between O and S atomic layers, giving rise to an intrinsically asymmetric structure along the out-of-plane direction. In comparison, the BSe monolayer crystallizes in a hexagonal honeycomb lattice composed of alternating B and Se atoms, as displayed in Fig. 1(b). Structural optimization yields equilibrium lattice constants of *a*_HfSO_ = 3.30 Å and *a*_BSe_ = 3.23 Å, respectively. These values agree well with previously reported theoretical results,^36,51^ confirming the reliability of the present computational approach. The electronic band structures of the isolated HfSO and BSe monolayers were subsequently calculated using both the PBE and HSE06 functionals, and the corresponding results are presented in Fig. 1. For the Janus HfSO monolayer, the calculated band structures reveal an indirect semiconducting nature, with the valence-band maximum (VBM) and conduction-band minimum (CBM) located at different high-symmetry *M* and *Γ* points in the Brillouin zone (BZ). Employing HSE06 increases the calculated HfSO gap from 1.23 eV at the PBE level to 2.03 eV. BSe likewise shows an indirect semiconducting character, with the predicted gap increasing from 2.62 eV using PBE to 3.46 eV when HSE06 is applied. As expected, the PBE functional underestimates the band-gap values due to its well-known limitations in describing exchange–correlation interactions. Nevertheless, both functionals predict the same qualitative electronic characteristics for the two monolayers.

**Fig. 1:**
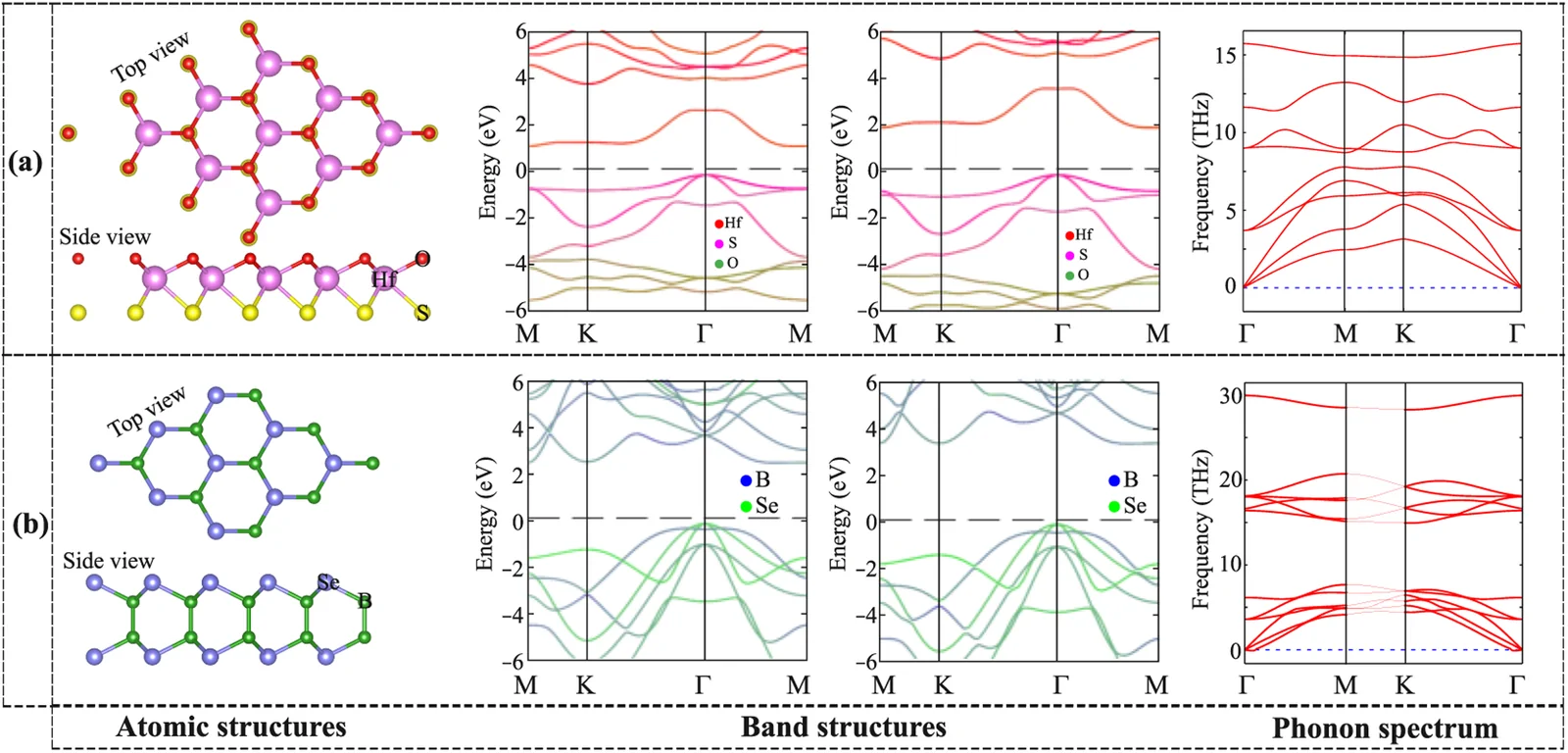
Atomic configurations, electronic band dispersions, and phonon spectra of (a) Janus HfSO and (b) BSe monolayers, shown from left to right. The O, Hf, and S atoms are depicted by red, violet, and yellow spheres, respectively, whereas B and Se are indicated by green and cyan markers.

To further evaluate the dynamical stability of the constituent monolayers, the phonon dispersion spectra of HfSO and BSe were calculated along the high-symmetry directions of the BZ. The phonon spectra of both systems exhibit no imaginary frequencies throughout the entire BZ, indicating that the optimized structures correspond to dynamically stable configurations. This result confirms that both HfSO and BSe monolayers can maintain their structural integrity against small lattice perturbations. Each monolayer exhibits the three acoustic branches characteristic of a two-dimensional lattice: longitudinal (LA), transverse (TA), and flexural out-of-plane (ZA) modes. Near *Γ*, the flexural ZA mode follows an approximately quadratic dispersion, reflecting the bending dynamics inherent to atomically thin sheets.

Based on the optimized monolayers, a BSe/HfSO van der Waals heterostructure was constructed by vertically stacking the BSe and HfSO layers. Owing to the intrinsic out-of-plane asymmetry of the Janus HfSO monolayer, two distinct interface configurations were considered depending on the surface facing the BSe layer, namely the S-terminated (S-face) and O-terminated (O-face) structures. For each termination, three different stacking arrangements were examined by varying the relative atomic registries of the constituent layers, as illustrated in Fig. 2. To build the BSe/HfSO heterostructure, we used a (1 × 1) unit cell of the BSe layer to match with a (1 × 1) unit cell of the HfSO layer. The lattice constant of the BSe/HfSO heterostructure is calculated to be 3.27 Å, which is the average of those of the constituent monolayers. This indicates that the HfSO layer is subjected to a 0.91% compressive strain, while the BSe layer is subjected to a 1.24% tensile strain. The overall lattice mismatch is sufficiently small and has a negligible effect on the intrinsic properties of the heterostructure. Furthermore, for the BSe/HfSO heterostructure, to identify the most stable atomic arrangement of the heterostructure, we evaluated three typical high-symmetry stacking registries. The calculating the total energies of these specific high-symmetry registries is a well-established and sufficient approach to determining the global lowest-energy stacking for our subsequent electronic structure calculations.^65,66^ For the AC registry, B sites in the BSe sheet are vertically aligned with Hf sites in HfSO, while the terminating O or S atoms lie beneath the centers of the B–Se hexagons. In the AB configuration, the Se atoms are vertically aligned with the Hf atoms, while the O (or S) atoms remain beneath the centers of the hexagonal rings. For the AA stacking arrangement, the Se and B atoms of the BSe layer are situated directly above the Hf and O (or S) atoms of the HfSO layer, respectively. The interlayer distances between the BSe and HfSO layers in their corresponding heterostructure are summarized in Table 1. Furthermore, the binding energy per unit interface area can also be defined as:1
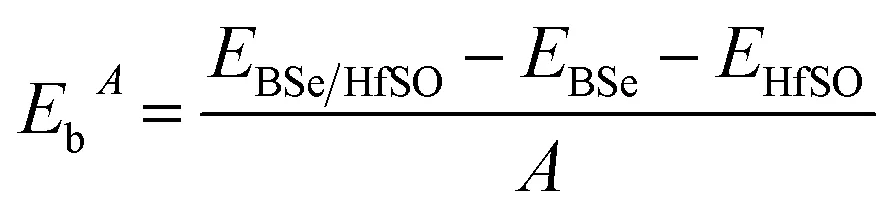
where *A* represents the interfacial area of the heterostructure, *E*_BSe/HfSO_ denotes the total energy of the fully relaxed BSe/HfSO heterostructure, while *E*_BSe_ and *E*_HfSO_ are the total energies of the isolated BSe and HfSO monolayers, respectively, calculated using the same computational parameters and supercell. A negative value of *E*_b_ indicates that the formation of the heterostructure is energetically favorable. The calculated binding energies of all six stacking configurations are negative, demonstrating that the formation of the BSe/HfSO heterostructure is energetically favorable. Interestingly, the binding energy strongly depends on both the stacking registry and the terminating surface of the Janus HfSO layer. The magnitude of the calculated binding energy is comparable to those reported for other representative two-dimensional vdW heterostructures. For example, an interfacial binding energy of approximately −20 meV Å^−2^ has been reported for the NbS_2_/BSe heterostructure, while binding energies ranging from −0.093 to −0.114 eV and from −0.178 to −0.194 eV have been obtained for HfSO/MoS_2_ and HfSeO/MoSe_2_ heterostructures, respectively.^53^ Among the six considered configurations, the AC-stacked O-terminated BSe/HfSO heterostructure is identified as the most energetically favorable configuration.

**Fig. 2:**
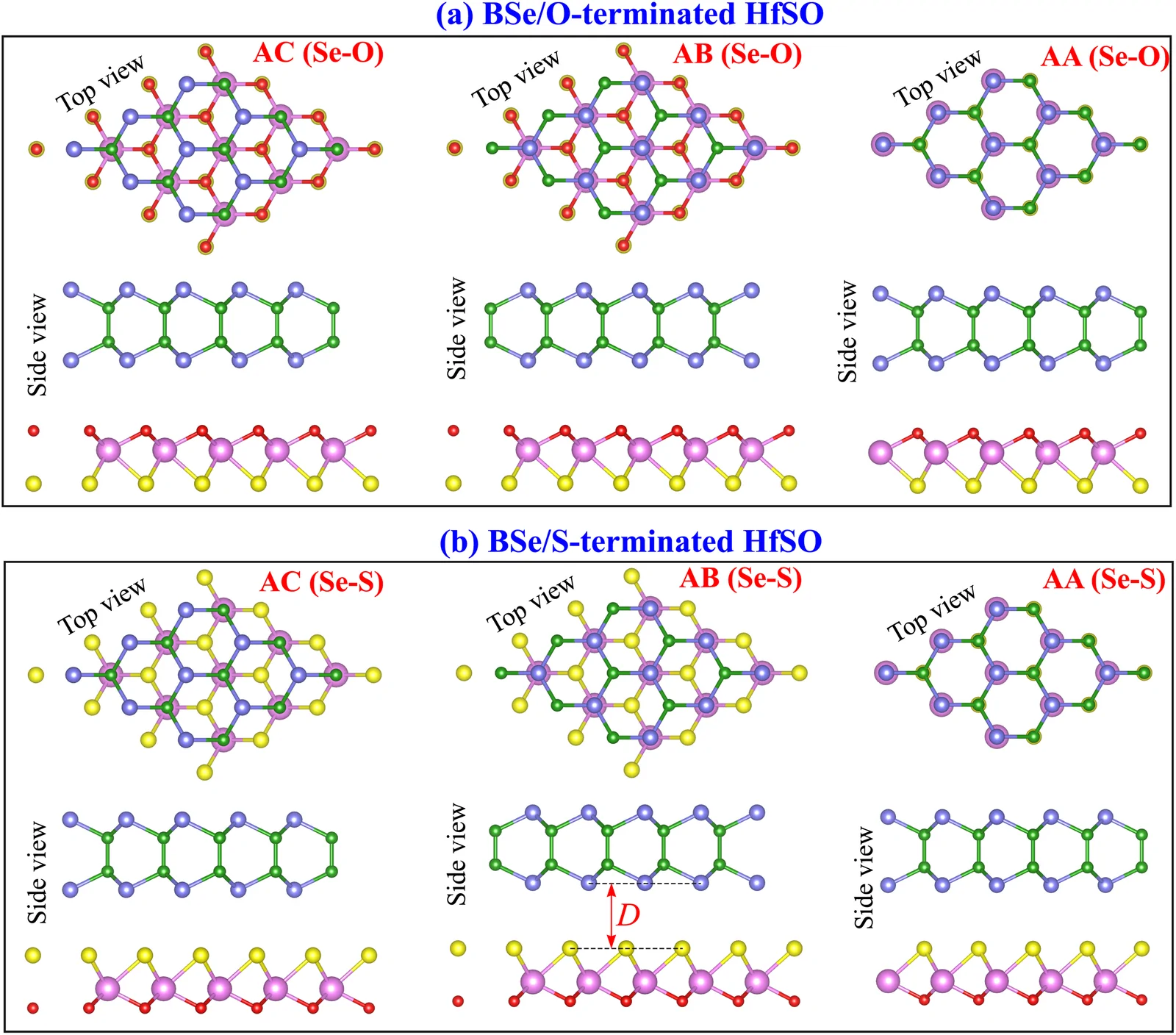
Optimized atomic structures of (a) BSe/HfSO with O-terminated surface and (b) BSe/HfSO with S-terminated surface for different stacking arrangements.

**Table 1:** Interlayer distances (*d*), binding energy (*E*_b_), band gap (*E*_g_), and contact type of the BSe/HfSO heterostructure

Heterostructure	Stacks	*d* (Å)	*E*_b_ (meV Å^−2^)	*E*_g_ (eV)	Contact
BSe/HfSO with O-terminated surface	AC	3.59	−28.64	1.03	Type-II
	AB	3.62	−24.25	1.08	Type-II
	AA	3.62	−25.32	1.09	Type-II
BSe/HfSO with S-terminated surface	AC	3.35	−16.53	0.12	Type-II
	AB	3.35	−17.46	0.12	Type-II
	AA	3.35	−16.78	0.12	Type-II

To gain further insight into the interfacial contact characteristics, the electronic band structures of the BSe/HfSO heterostructures with both O- and S-terminated interfaces were calculated for the three considered stacking configurations. Fig. 3 shows that stacking produces band dispersions that remain largely consistent with the individual-layer electronic structures, suggesting only limited modification of the intrinsic states upon heterostructure formation. Such behavior is a typical feature of vdW heterostructures. Despite the preservation of the band structures, the formation of the heterostructure results in a pronounced reduction of the band gap relative to those of the isolated monolayers. For the O-terminated interface, the calculated band gaps range from 1.03 to 1.09 eV for the three stacking configurations, which are substantially smaller than those of the constituent monolayers. In contrast, the S-terminated heterostructures exhibit much narrower band gaps of 0.12 eV regardless of the stacking arrangement, demonstrating that the interface termination has a significant influence on the electronic structure. The consistently smaller band gaps of the S-terminated configurations suggest a stronger interlayer electronic coupling than that of the O-terminated counterparts. Similar stacking-dependent modulation of interlayer van der Waals coupling has been reported for polar Janus SMoSe/SWSe heterostructures.^26^

**Fig. 3:**
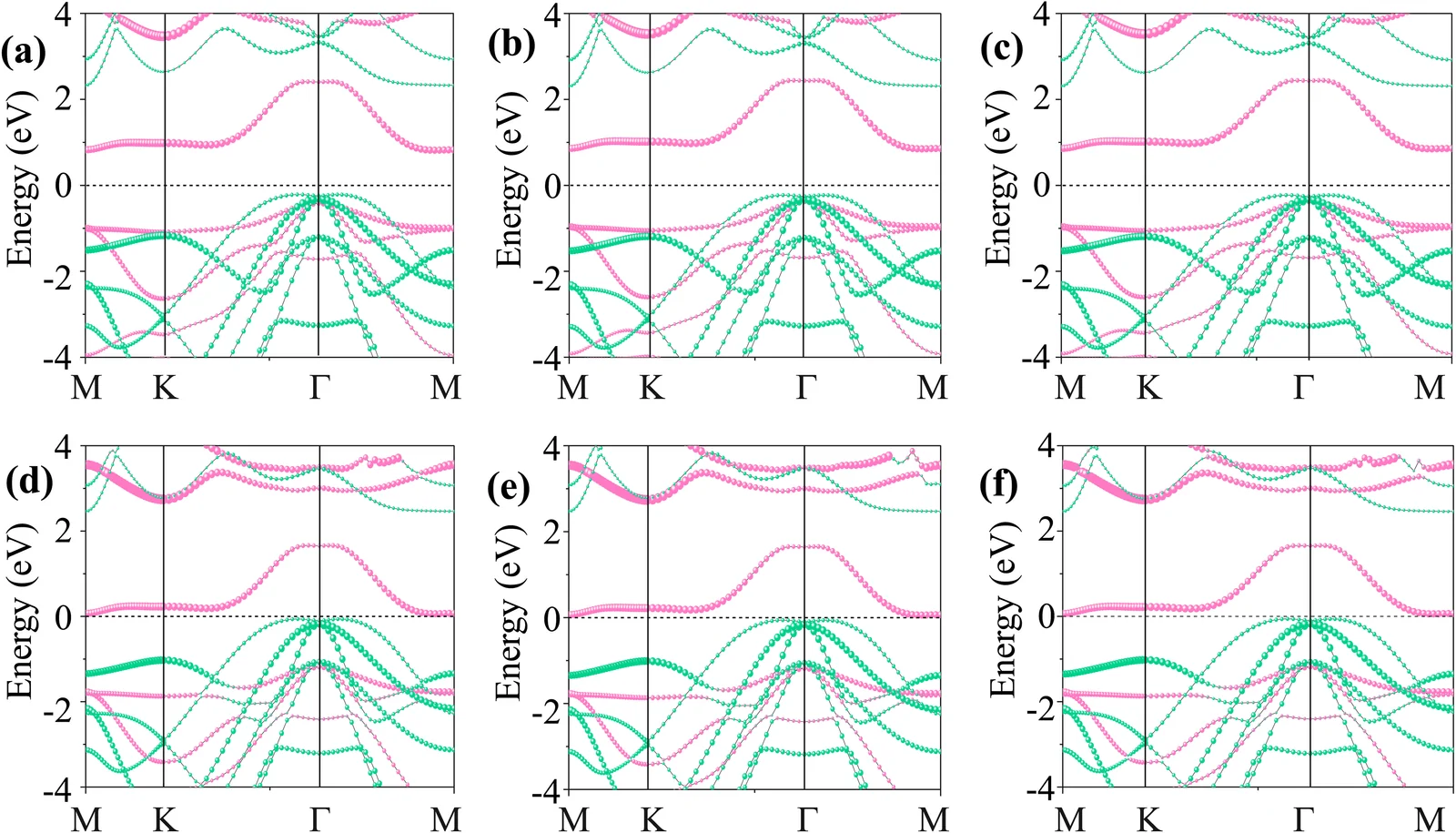
Layer-resolved electronic band structures calculated for BSe/HfSO interfaces with O- and S-terminated surfaces: (a) AC (Se–O), (b) AB (Se–O), (c) AA (Se–O), (d) AC (Se–S), (e) AB (Se–S), and (f) AA (Se–S) configurations. The HfSO- and BSe-derived states are distinguished by violet and green curves, respectively.

More importantly, all energetically favorable BSe/HfSO heterostructures exhibit a staggered type-II band alignment, in which the VBM and CBM originate from different constituent layers. For all stacking configurations, the VBM is located along the *K*–*M* high-symmetry path and is primarily derived from the BSe monolayer, whereas the CBM resides at the *Γ* point and is mainly contributed by the HfSO monolayer. Because the VBM and CBM are predominantly localized on different constituent layers, the type-II band alignment may facilitate spatial separation of photoexcited electrons and holes. However, the actual exciton dissociation and carrier recombination dynamics require further investigation. In addition, when the S-terminated surface faces the BSe monolayer, both the valence- and conduction-band edges shift closer to the Fermi level than those of the O-terminated counterpart, indicating a stronger interlayer electronic coupling. This enhanced coupling results in a more pronounced band-gap reduction. These results demonstrate that the electronic band gap of the BSe/HfSO heterostructure can be effectively tailored by engineering both the interface termination and the stacking configuration.

To provide further evidence for the type-II band alignment, the projected density of states (PDOS), layer-resolved band structures calculated using the HSE06 hybrid functional, and the corresponding schematic band-edge alignments were examined. The results are summarized in Fig. 4. As shown in Fig. 4(a), the states near the VBM are predominantly contributed by the hybridized B-2p and Se-2p orbitals of the BSe monolayer, whereas the CBM is mainly derived from the Hf-d orbitals of the HfSO layer. These layer-resolved states provide additional evidence that the BSe/HfSO system adopts a staggered type-II band configuration. Because PBE is known to underestimate semiconductor band gaps, complementary calculations were carried out with the HSE06 hybrid functional. The HSE06 calculations provide an independent check of the electronic structure and band alignment predicted for BSe/HfSO, as shown in Fig. 4(b). Compared with the PBE results, the most noticeable difference introduced by the HSE06 functional is the upward shift of the CBM associated with the HfSO layer toward higher energies, while the band dispersion remains nearly unchanged. Consequently, the gap widens, whereas the staggered type-II configuration remains intact. Despite the quantitative increase in the gap obtained with HSE06, neither the overall dispersion nor the type-II character changes appreciably, supporting the reliability of the PBE-based interpretation. The schematic alignment in Fig. 4(c) likewise shows that the VBM is associated with BSe, whereas the CBM is derived primarily from HfSO layer. The spatial localization of the VBM and CBM on different layers provides an energetic basis for spatially separated electron and hole localization following photoexcitation. Such a type-II band alignment may facilitate charge separation. However, the efficiency of exciton dissociation, carrier lifetime, recombination, and extraction cannot be established from the present ground-state electronic-structure calculations alone.

**Fig. 4:**
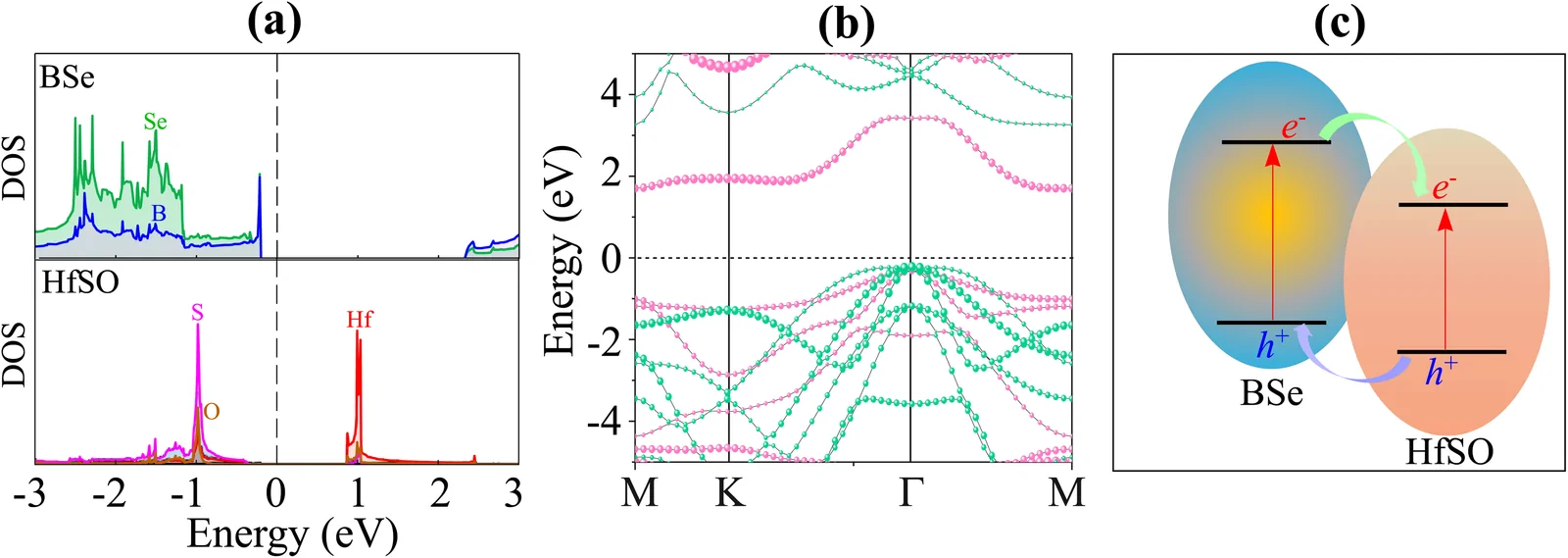
(a) Projected density of states (PDOS), (b) HSE06 projected band structures and (c) band alignment scheme of the BSe/HfSO heterostructure for the most energetically favorable stacking configuration.

The stability of the lowest-energy BSe/HfSO configuration was examined using both phonon calculations and AIMD, with the resulting data summarized in Fig. 5. As shown in Fig. 5(a), no imaginary phonon modes are observed throughout the entire Brillouin zone, demonstrating the dynamical stability of the optimized heterostructure. This result indicates that the vertically stacked BSe/HfSO configuration corresponds to a stable local minimum on the potential-energy surface and is therefore resistant to spontaneous lattice distortions. The thermal stability of the heterostructure was further examined by AIMD simulations at 300 K within the NVT ensemble. As depicted in Fig. 5(b), the instantaneous temperature fluctuates around the room temperature of 300 K, while the total energy exhibits only small oscillations throughout the simulation without any abrupt variation or continuous energy drift. The final AIMD structure retains its original topology, with neither significant reconstruction nor bond rupture or delamination detected. These results confirm that the BSe/HfSO heterostructure remains thermally stable.

**Fig. 5:**
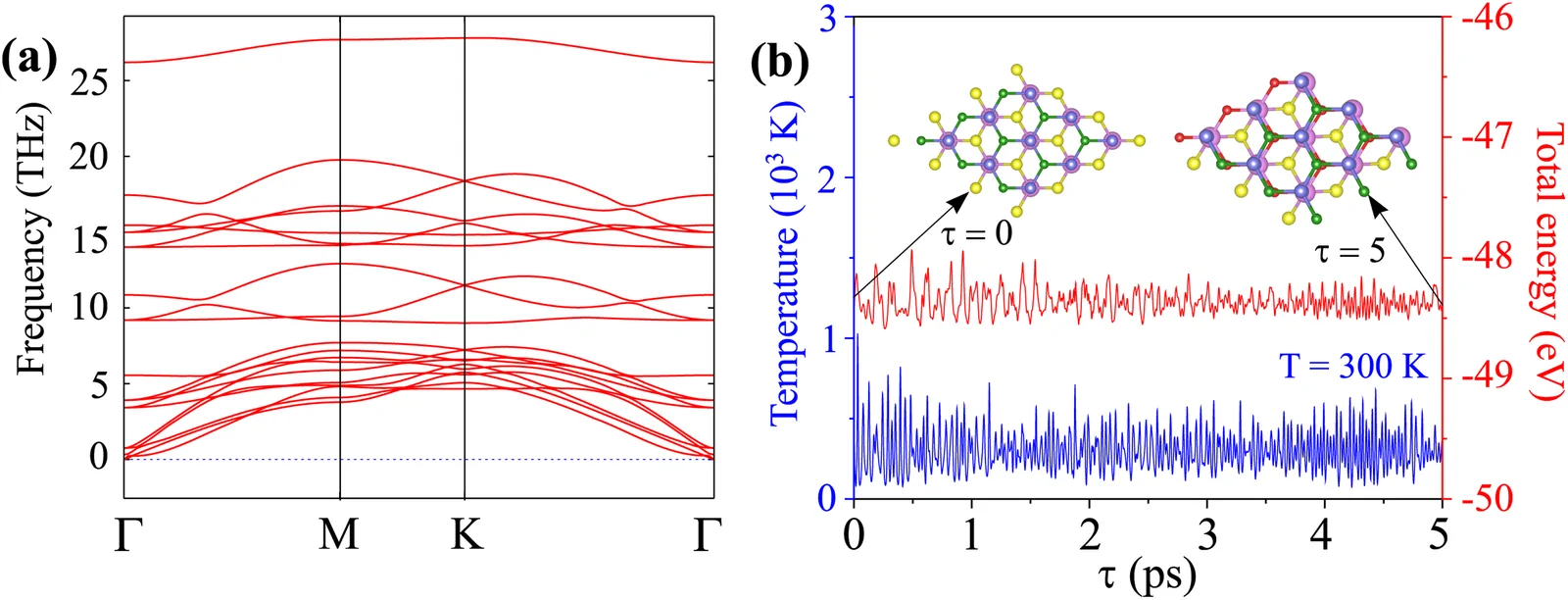
(a) Phonon spectrum and (b) temperature and total-energy evolutions obtained from AIMD simulations for the AC-stacked O-terminated BSe/HfSO heterostructure.

To gain further insight into the interfacial interaction and mechanical stability of the BSe/HfSO heterostructure, the electron localization function (ELF) and elastic constants were analyzed, as shown in Fig. 6. The ELF distribution presented in Fig. 6(a) reveals a pronounced localization of electrons around the constituent atoms, whereas a relatively low ELF value is observed in the interfacial region between the BSe and HfSO layers. This feature indicates that no covalent bonds are formed across the interface and confirms that the two monolayers are coupled predominantly through weak vdW interactions. At the same time, the absence of significant electron accumulation or depletion between the adjacent layers suggests that the intrinsic chemical bonding within each monolayer is well preserved after heterostructure formation. Fig. 6(b) compares the calculated elastic constants of BSe/HfSO with those obtained for the two isolated layers. The calculated elastic constants fulfill the Born stability requirements for a two-dimensional hexagonal lattice, namely *C*_11_ > 0, *C*_66_ > 0 and *C*_11_^2^ − *C*_12_^2^ > 0, thereby establishing mechanical stability. Moreover, relative to the isolated sheets, BSe/HfSO exhibits larger *C*_*ij*_ values, indicating that interlayer assembly increases the in-plane mechanical stiffness. The in-plane elastic response was further quantified through Young's modulus, which was obtained from the calculated elastic constants according to:2
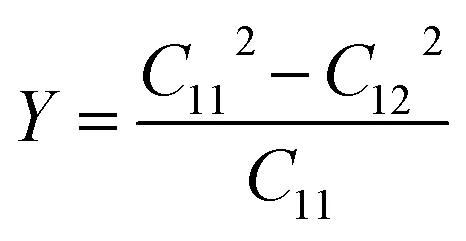


**Fig. 6:**
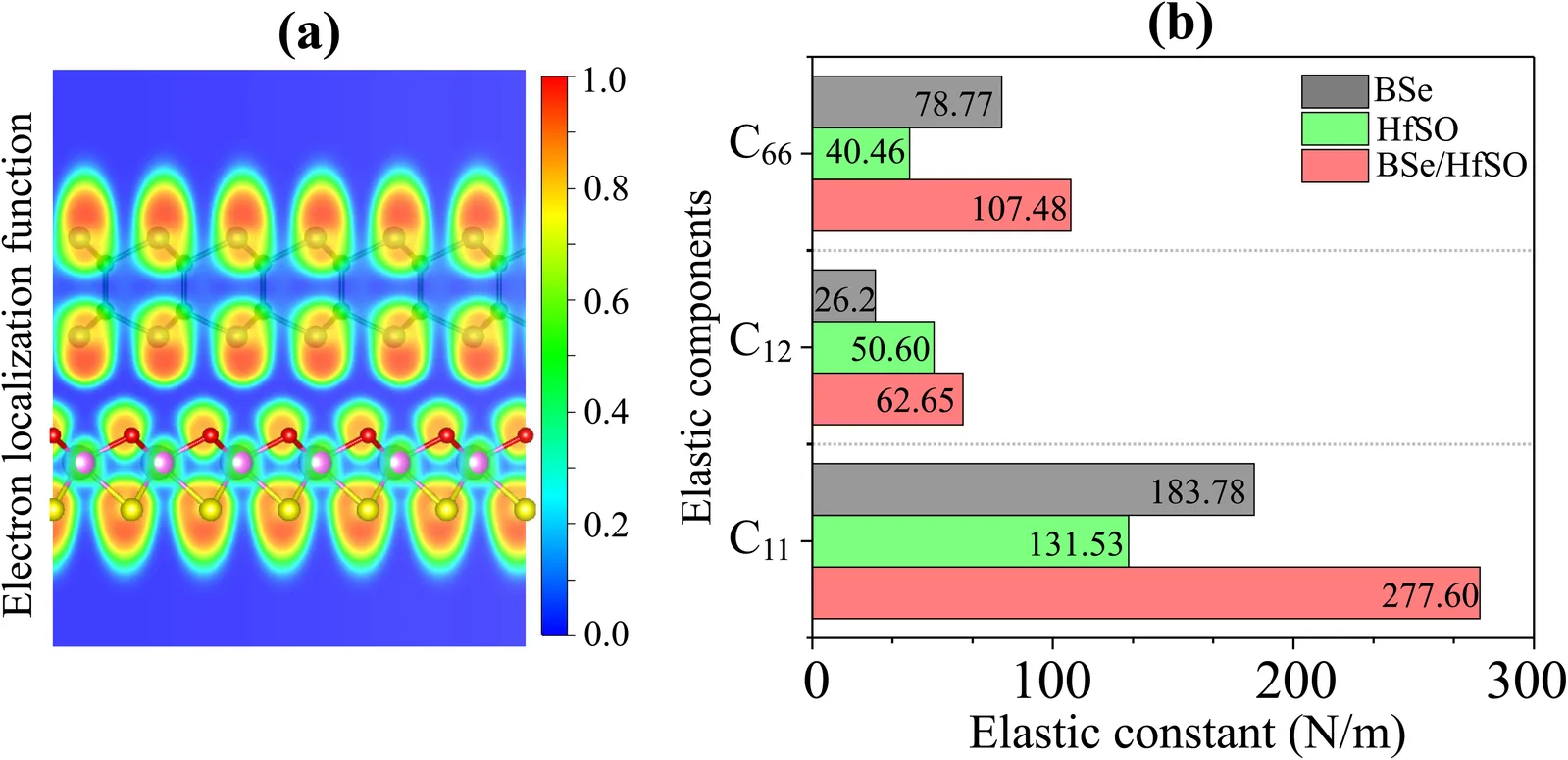
(a) Spatial distribution of the electron localization function (ELF) and (b) calculated elastic constants for the AC-stacked O-terminated BSe/HfSO heterostructure, together with the corresponding values of the isolated monolayers.

The resulting in-plane Young's modulus is 243.47 N m^−1^ for BSe/HfSO, compared with 180.04 N m^−1^ and 112.06 N m^−1^ for isolated BSe and HfSO, respectively. This result suggests that vertical stacking effectively enhances the mechanical rigidity of the heterostructure.

The interfacial charge-transfer process was further examined by comparing the plane-averaged electrostatic potentials and work functions of the isolated layers with those of the heterostructure. The work function (*Φ*) measures the energy difference between the Fermi level and the vacuum reference, and is defined as:

3 *Φ* = *E*_vac_ − *E*_F_,

where *E*_vac_ and *E*_F_ represent the vacuum and Fermi-energy levels, respectively. Since the work function directly reflects the electron-emission capability of a material, it also serves as an important indicator for predicting the direction of interfacial charge transfer upon the formation of a vdW heterostructure. Electrons tend to transfer spontaneously from the material with a lower work function to that with a higher work function until thermodynamic equilibrium is established, resulting in the formation of an intrinsic built-in electric field across the interface. As shown in Fig. 7, the work functions calculated using the HSE06 hybrid functional are 6.38, 6.30 and 7.70 eV for the BSe, O-terminated and S-terminated HfSO monolayers, respectively. The considerably lower work function of the BSe monolayer indicates a weaker electron-binding capability than that of HfSO layer. Upon contact, charge redistribution is therefore expected to proceed from BSe toward HfSO until a common Fermi-level equilibrium is established. After stacking, the calculated work function becomes 7.03 and 6.56 eV for the HfSO and BSe surfaces, respectively. This intermediate value is consistent with interfacial charge redistribution and electrostatic equilibration after contact, supporting the formation of an intrinsic interfacial electric field. The interfacial redistribution was further quantified through the charge-density difference, which was evaluated as:

4 Δ*ρ* = *ρ*_BSe/HfSO_ − *ρ*_BSe_ − *ρ*_HfSO_

where *ρ*_BSe/HfSO_, *ρ*_BSe_ and *ρ*_HfSO_ denote the charge densities of the heterostructure, isolated BSe monolayer, and isolated HfSO monolayer, respectively. The charge densities of the isolated monolayers were calculated using the same atomic positions as those in the optimized heterostructure. The calculated charge-density difference is illustrated in Fig. 7(d), where the red and green isosurfaces represent electron accumulation and electron depletion, respectively. It can be clearly observed that electron accumulation mainly occurs around the HfSO layer, whereas electron depletion is predominantly distributed near the BSe layer. The resulting charge-density-difference profile reveals interfacial charge redistribution, with electron accumulation predominantly around the HfSO layer and electron depletion mainly around the BSe layer. This redistribution pattern is consistent with the work-function analysis, which indicates that electrons are energetically favored to redistribute from the lower-work-function BSe layer toward the higher-work-function HfSO layer until Fermi-level equilibrium is established. The interfacial charge redistribution consequently generates an interfacial dipole and an associated intrinsic built-in electric field directed from the electron-depleted BSe layer toward the electron-accumulated HfSO layer. It should be noted that the direction of the electrostatic electric field should be distinguished from the direction of the force acting on electrons. Because electrons carry a negative charge, the electrostatic force acting on an electron is opposite to the direction of the electric field. Therefore, the intrinsic built-in electric field identified here should not be interpreted as directly driving photoexcited electrons from BSe toward HfSO. Instead, the calculated type-II band alignment provides an energetic basis for spatially separated carrier localization, with the VBM predominantly localized on the BSe layer and the CBM mainly derived from the HfSO layer. Such spatial separation of the band-edge states may facilitate the separation of photoexcited electrons and holes.

**Fig. 7:**
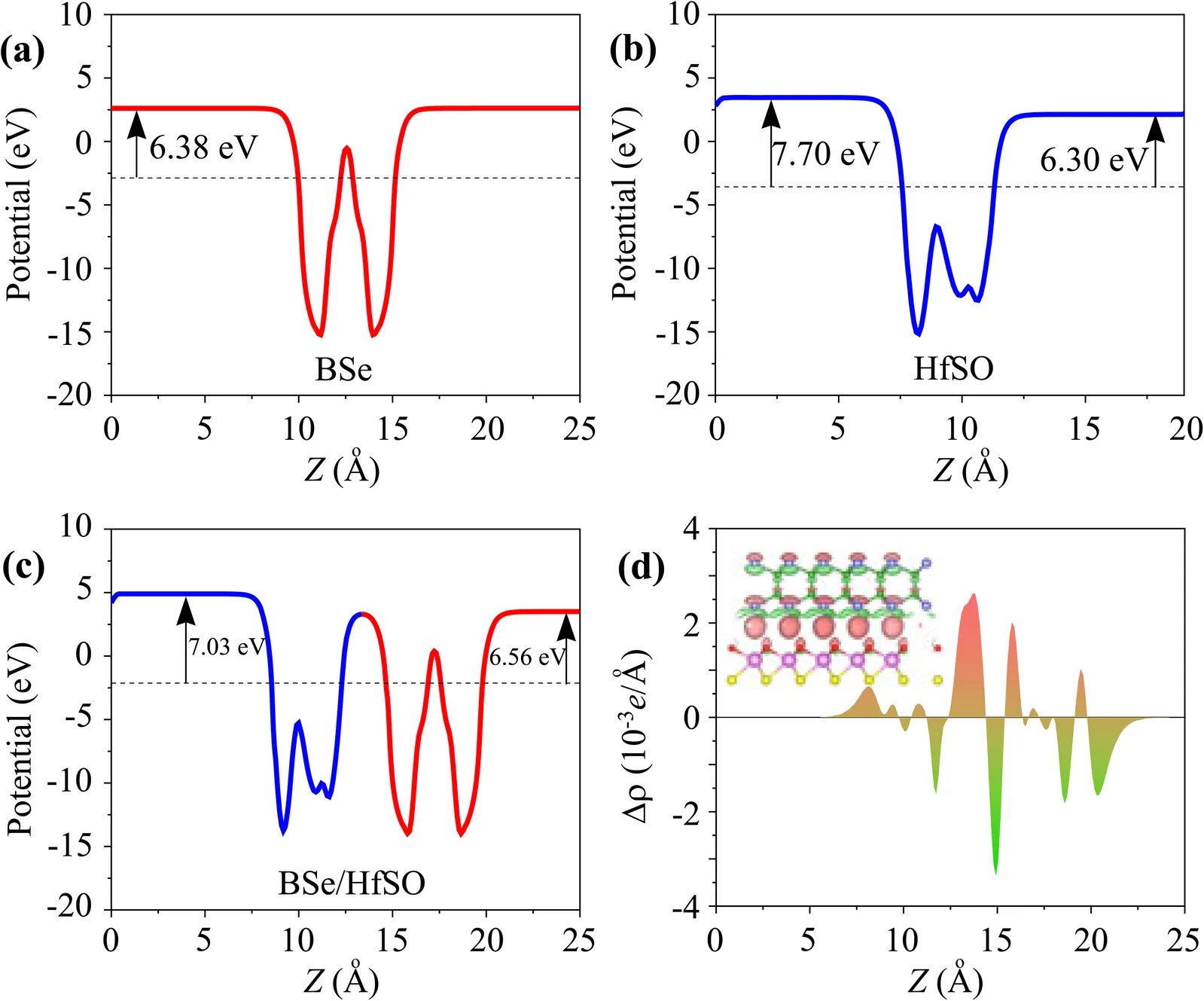
Plane-averaged electrostatic potentials calculated for (a) isolated BSe, (b) isolated HfSO, and (c) the AC-stacked O-terminated BSe/HfSO heterostructure. Panel (d) presents the corresponding charge-density-difference profile along the out-of-plane direction. The inset depicts the three-dimensional charge redistribution, with red and green isosurfaces denoting electron accumulation and depletion, respectively.

To evaluate the optical response of the BSe/HfSO heterostructure, the frequency-dependent optical absorption coefficient was calculated from the complex dielectric function. The dielectric function can be expressed as:

5 *ε*(*ω*) = *ε*_1_(*ω*) + *iε*_2_(*ω*)

where *ε*_1_(*ω*) and *ε*_2_(*ω*) are the real and imaginary parts of the dielectric function, respectively. *ω* is the angular frequency of the incident photon. Since the dielectric response obtained from a periodic slab calculation depends on the vacuum spacing, the calculated optical response was normalized with respect to the effective physical thickness of the BSe/HfSO heterostructure. Here, *d*_eff_ = 9.88 Å represents the effective physical thickness of the heterostructure, while the vacuum region introduced to avoid interactions between periodically repeated slabs was excluded. The optical absorption coefficient was then determined by:6
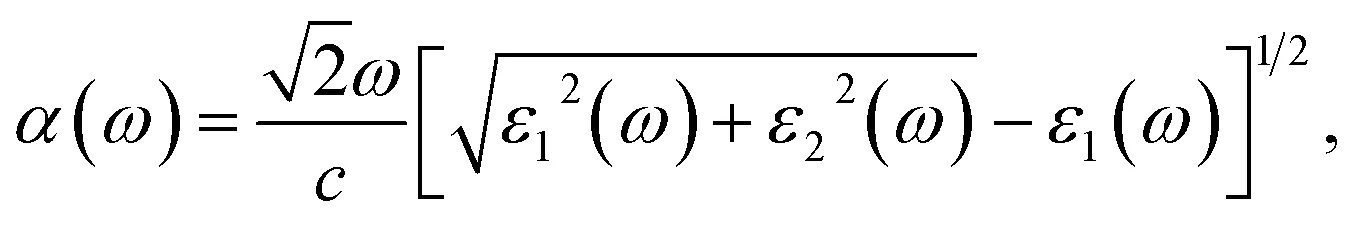


Fig. 8 compares the calculated absorption spectra of isolated BSe, isolated HfSO, and the BSe/HfSO heterostructure. Relative to the individual sheets, BSe/HfSO displays substantially stronger absorption across a wide spectral interval, with particularly pronounced enhancement between 1.60 and 3.20 eV. The absorption onset also moves to lower photon energies, producing a clear red shift compared with the isolated components. This spectral shift is consistent with the gap narrowing caused by vdW coupling, which allows optical excitations to begin at lower energies. At higher photon energies, the heterostructure absorption coefficient increases sharply, attaining values of approximately 10^5^ cm^−1^, which are significantly higher than those of the isolated BSe monolayer and comparable to or even exceeding those of the HfSO monolayer over a wide portion of the visible spectrum. Furthermore, the strong absorption overlapping with the solar spectrum suggests that the BSe/HfSO heterostructure can effectively harvest visible sunlight. It should be noted that excitonic effects were not explicitly included in the present calculations, as the optical response was evaluated within the independent-particle PBE framework. Therefore, exciton binding effects and excitonic resonances are not captured in the calculated absorption spectrum. Nevertheless, the PBE-based results provide useful insights into the optical absorption characteristics of the BSe/HfSO heterostructure within the adopted computational framework.

**Fig. 8:**
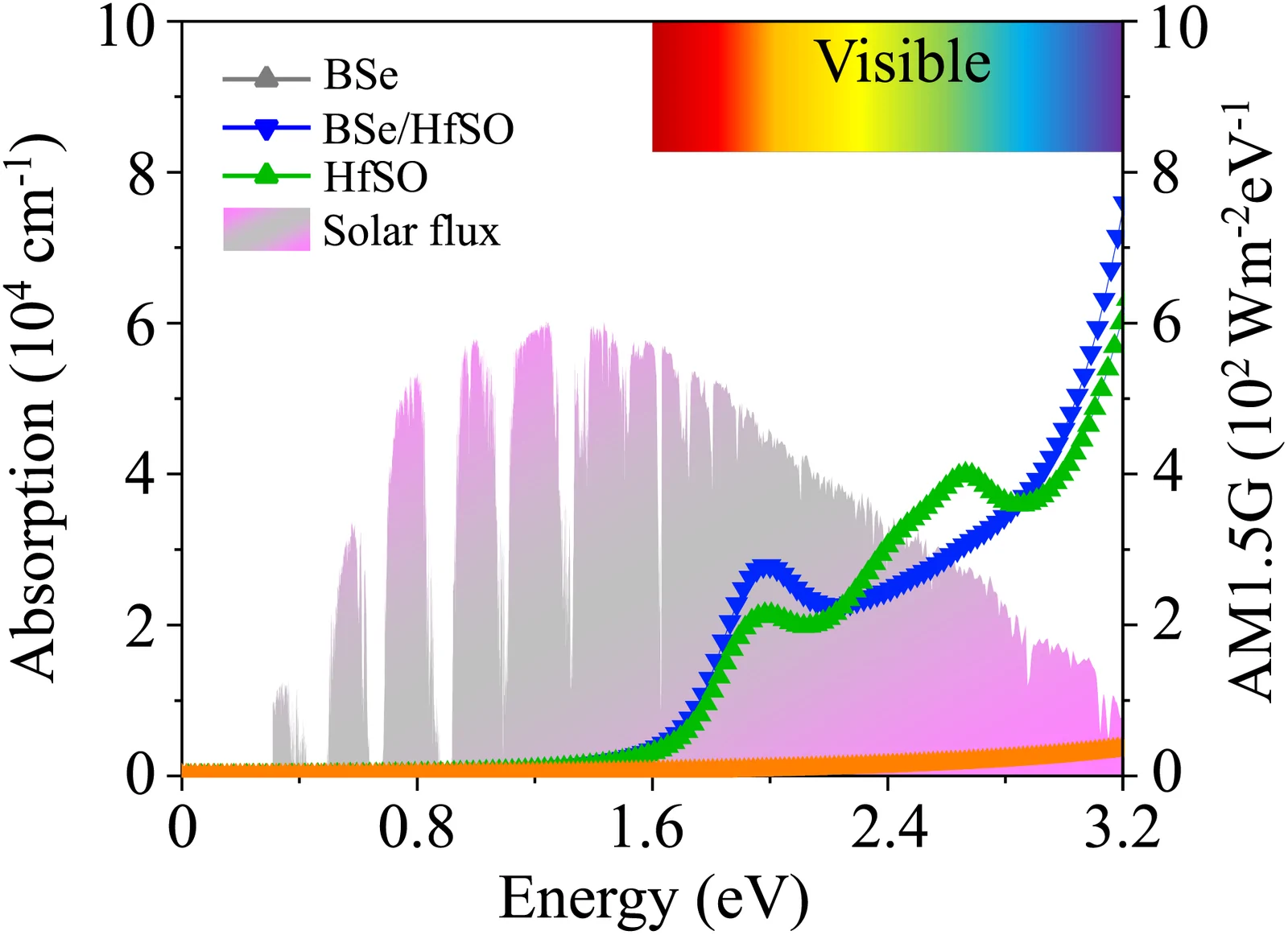
Calculated optical absorption spectra of the BSe and HfSO monolayers together with the AC-stacked O-terminated BSe/HfSO heterostructure for *E*‖*z* polarization using PBE functional. The AM1.5G solar spectrum and the visible-light region are included for comparison.

The photovoltaic capability of BSe/HfSO was further quantified by estimating its power-conversion efficiency (PCE) through the Scharber model:^67^7
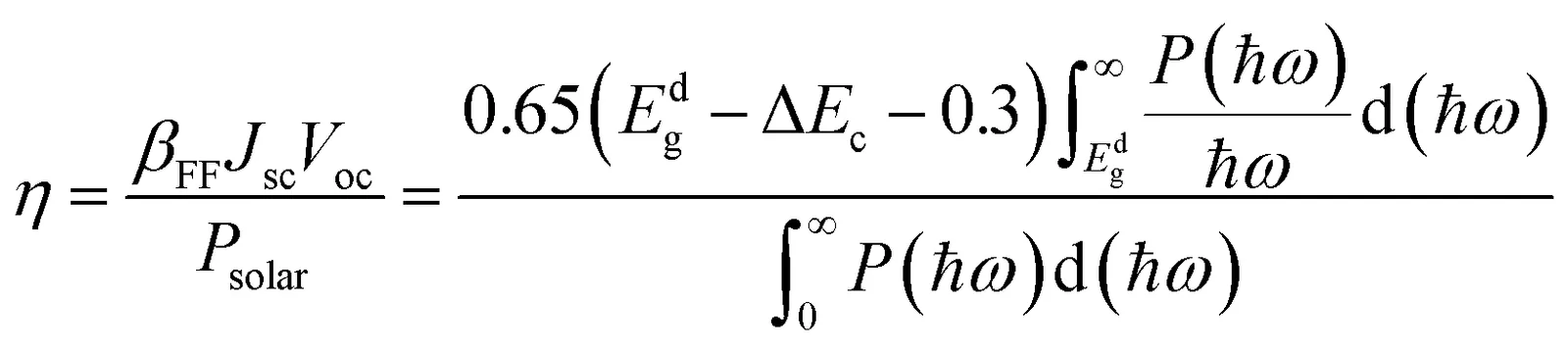
where *β*_FF_ is the fill factor, taken as 0.65, *V*_oc_ denotes the open-circuit voltage, *J*_sc_ is the short-circuit current density, and *P*_solar_ represents the total incident solar power under the standard AM1.5G solar spectrum. The open-circuit voltage is estimated by

8 *V*_oc_ = *E*^d^_g_ − Δ*E*_c_ − 0.30

where *E*^d^_g_ is the band gap of the donor layer and Δ*E*_c_ is the conduction-band offset between the and acceptor. In this work, all these values are obtained from the PBE functional. In the BSe/HfSO heterostructure, the work-function difference and the calculated interfacial charge redistribution suggest that BSe serves as the electron-donor layer, whereas HfSO acts as the electron-acceptor layer. Accordingly, *E*^d^_g_ = 3.48 eV corresponds to the band gap of the BSe layer, while Δ*E*_c_ = 1.77 eV is determined from the relative CBM positions of the BSe and HfSO layers. The short-circuit current density is evaluated according to9
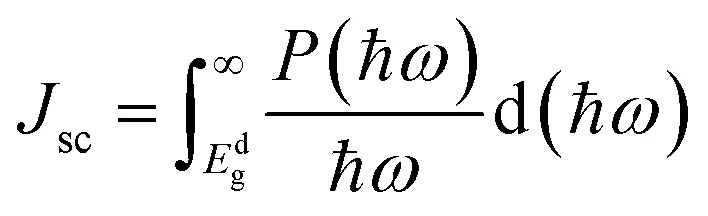
where *P*(*ℏω*) denotes the photon flux of the AM1.5G solar spectrum. The obtained *J*_sc_ is 4.67 mA cm^−2^ and the theoretically estimated PCE of BSe/HfSO heterostructure is 4.27% based on the Scharber model. The estimated PCE is within the range reported for several recently studied vdW heterostructures, including GaSe/SnS_2_ (4.0%),^68^ BAs/WSe_2_ (4.48%),^69^ HfSeO/MoSe_2_ (4.57%),^53^ GaN/MoSSe (2.8%),^70^ and MoS_2_/Si (5.23%).^71^ It should be emphasized that this value represents an idealized theoretical estimate within the Scharber-model framework, which assumes a fixed fill factor of 0.65 and an empirical voltage loss of 0.30 eV. In addition, the evaluation of *J*_sc_ from the AM1.5G photon flux assumes ideal photocurrent generation for photons above the donor band gap and does not explicitly account for incomplete photon absorption, exciton dissociation, non-radiative recombination, or carrier-collection losses. Therefore, the calculated PCE should not be interpreted as the experimentally achievable photovoltaic efficiency of the BSe/HfSO heterostructure.

The electronic tunability of the lowest-energy BSe/HfSO configuration was subsequently examined by applying an external electric field, as illustrated in Fig. 9(a). The external electric field was introduced using a sawtooth potential applied perpendicular to the heterostructure along the *z* direction. To investigate the effect of the external electric field on the electronic properties, the atomic positions were kept fixed at the optimized zero-field geometry. For each applied electric-field strength, self-consistent-field (SCF) calculations were subsequently performed, followed by band-structure calculations using the corresponding self-consistent charge density. The positive electric field is defined as pointing from the HfSO layer toward the BSe layer, whereas the negative field corresponds to the opposite direction. The electric-field strength was varied from −0.3 to +0.3 V Å^−1^ to investigate its influence on the electronic structure. To avoid artificial vacuum states and periodic-boundary effects, we carefully monitored the macroscopic electrostatic potential profile and charge density, ensuring the vacuum potential remained above the Fermi level at the cell boundaries. The corresponding evolution of the band gap as a function of the external electric field is presented in Fig. 9(b). The calculated results demonstrate that the electronic structure of BSe/HfSO is highly responsive to the applied field. Applying a negative field progressively narrows the gap, from about 0.65 eV at −0.3 V Å^−1^ without altering the type-II configuration. This behavior indicates that the intrinsic staggered band alignment is robust against negative electric fields. As the electric field is further increased in the positive direction, the band gap continues to increase slightly and reaches a nearly saturated value of approximately 1.20 eV. Once the positive field reaches roughly +0.1 V Å^−1^, the system switches from the staggered type-II arrangement to a type-I alignment. Such a transition originates from the electric-field-induced shift of the band edges, which modifies the relative energy positions of the BSe and HfSO layers, as depicted in Fig. 10. In addition, the observed modulation can be attributed to the Stark effect induced by the external electric field. Because charge transfer establishes an intrinsic interfacial field in BSe/HfSO, an external field can either reinforce or oppose this internal potential depending on its orientation. Changing the external-field direction therefore modifies the interfacial potential drop and shifts the relative band-edge energies, ultimately enabling the type-II/type-I conversion.

**Fig. 9:**
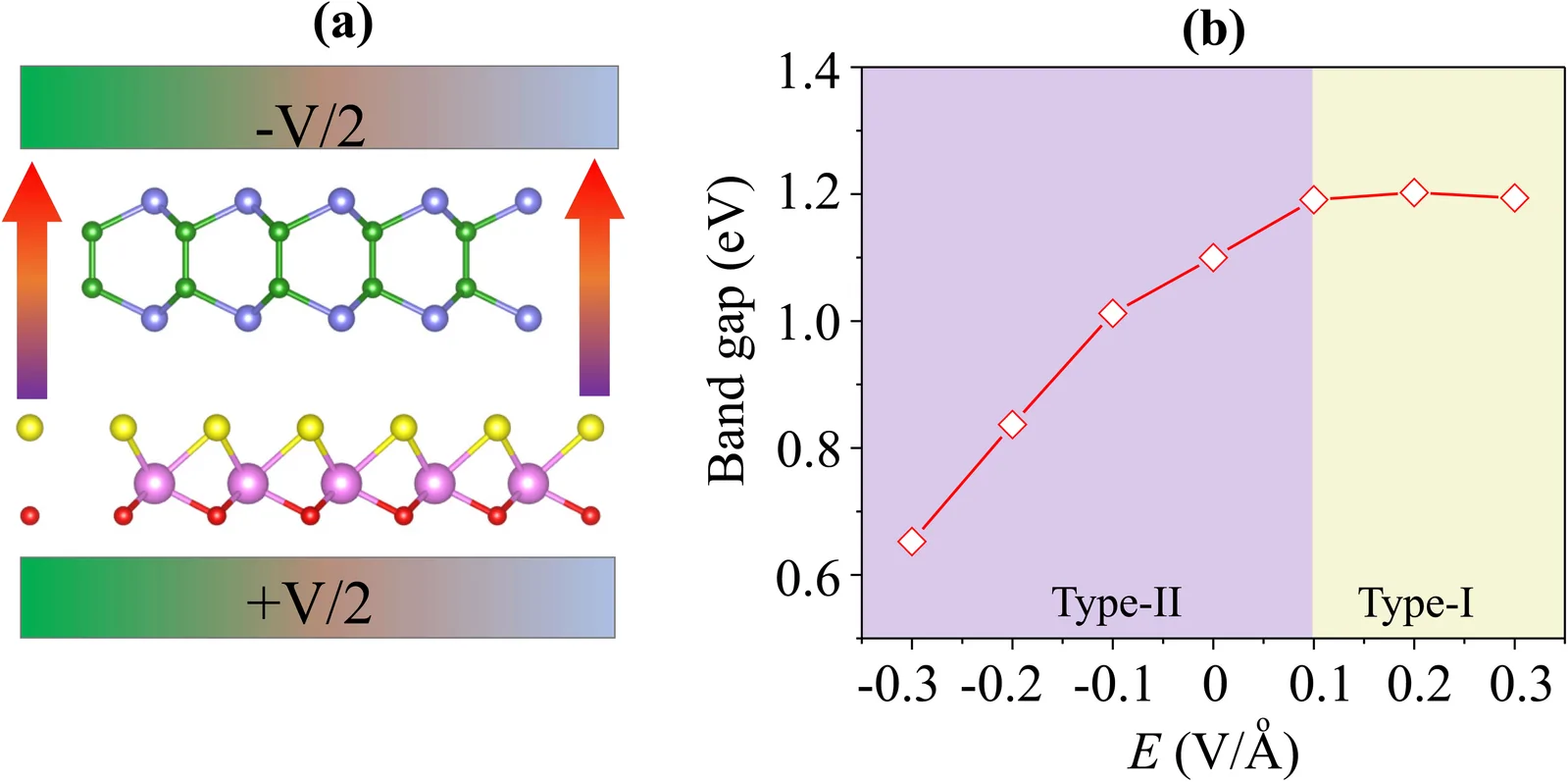
(a) Schematic representation of BSe/HfSO subject to an external perpendicular electric field. (b) PBE-calculated band-gap variation with electric-field strength of the AC-stacked O-terminated BSe/HfSO heterostructure.

**Fig. 10:**
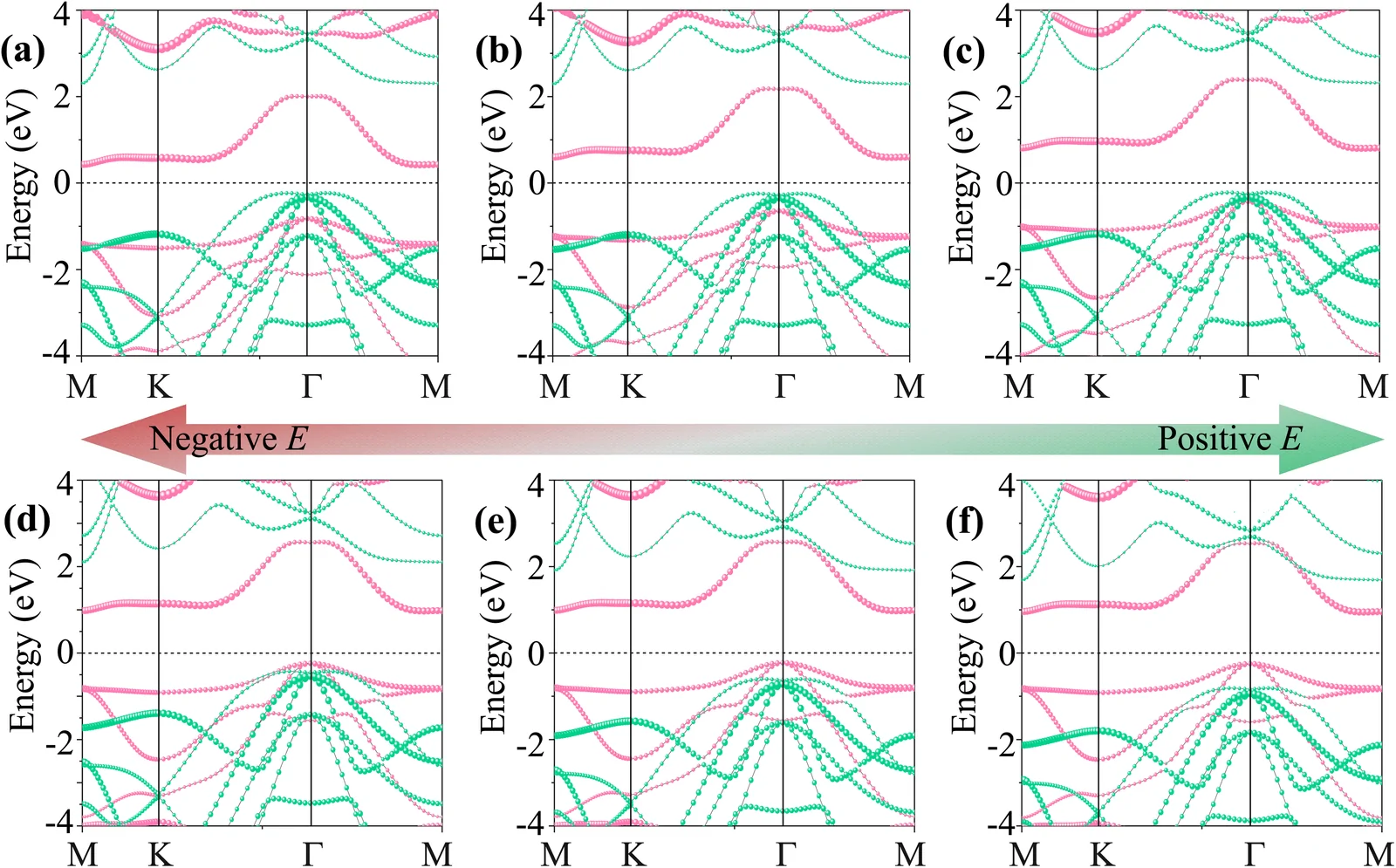
Layer-projected PBE band dispersions of the AC-stacked O-terminated BSe/HfSO heterostructure calculated under external electric fields of (a) −0.3, (b) −0.2, (c) −0.1, (d) +0.1, (e) +0.2, and (f) +0.3 V Å^−1^. Contributions from the HfSO and BSe layers are indicated by pink and cyan curves, respectively.

The origin of the field-dependent gap variation can be clarified by examining the layer-projected band structures calculated at different field strengths, as shown in Fig. 10. Layer-resolved bands show distinct field responses for BSe and HfSO, resulting in a gradual displacement of their respective band edges. Under negative electric fields [Fig. 10(a)–(c)], the CBM remains localized in the HfSO layer, whereas the VBM is still dominated by the BSe layer, indicating that the staggered type-II band alignment is preserved. Meanwhile, the relative energy separation between the CBM and VBM gradually decreases with increasing negative electric field, giving rise to the monotonic reduction of the band gap observed in Fig. 9(b). As the external electric field changes from negative to positive values, the band edges exhibit an opposite energy shift. In particular, under positive electric fields [Fig. 10(d)–(f)], the CBM associated with the HfSO layer is shifted upward relative to that of the BSe layer, resulting in a reconstruction of the band-edge alignment. At sufficiently positive fields, the VBM and CBM shift onto the same layer, converting the original staggered type-II configuration into a type-I alignment. This transition is fully consistent with the critical electric field of approximately +0.1 V Å^−1^ identified in Fig. 9(b). Furthermore, to verify the reliability of this field-induced transition, additional HSE06 calculations were performed at electric field of +0.1 V Å^−1^, as shown in Fig. 11. The HSE06 results predict that the HfSO/BSe heterostructure exhibits type-I band alignment at +0.1 V Å^−1^. The consistency between the PBE and HSE06 results further supports the predicted type-II to type-I transition induced by the external electric field. The evolution of the band-edge positions under external electric fields demonstrates the excellent electrostatic tunability of the BSe/HfSO heterostructure. Field-controlled switching between the two band configurations provides a means of regulating carrier separation and recombination, thereby expanding the potential functionality of the heterostructure in nanoelectronic and optoelectronic applications.

**Fig. 11:**
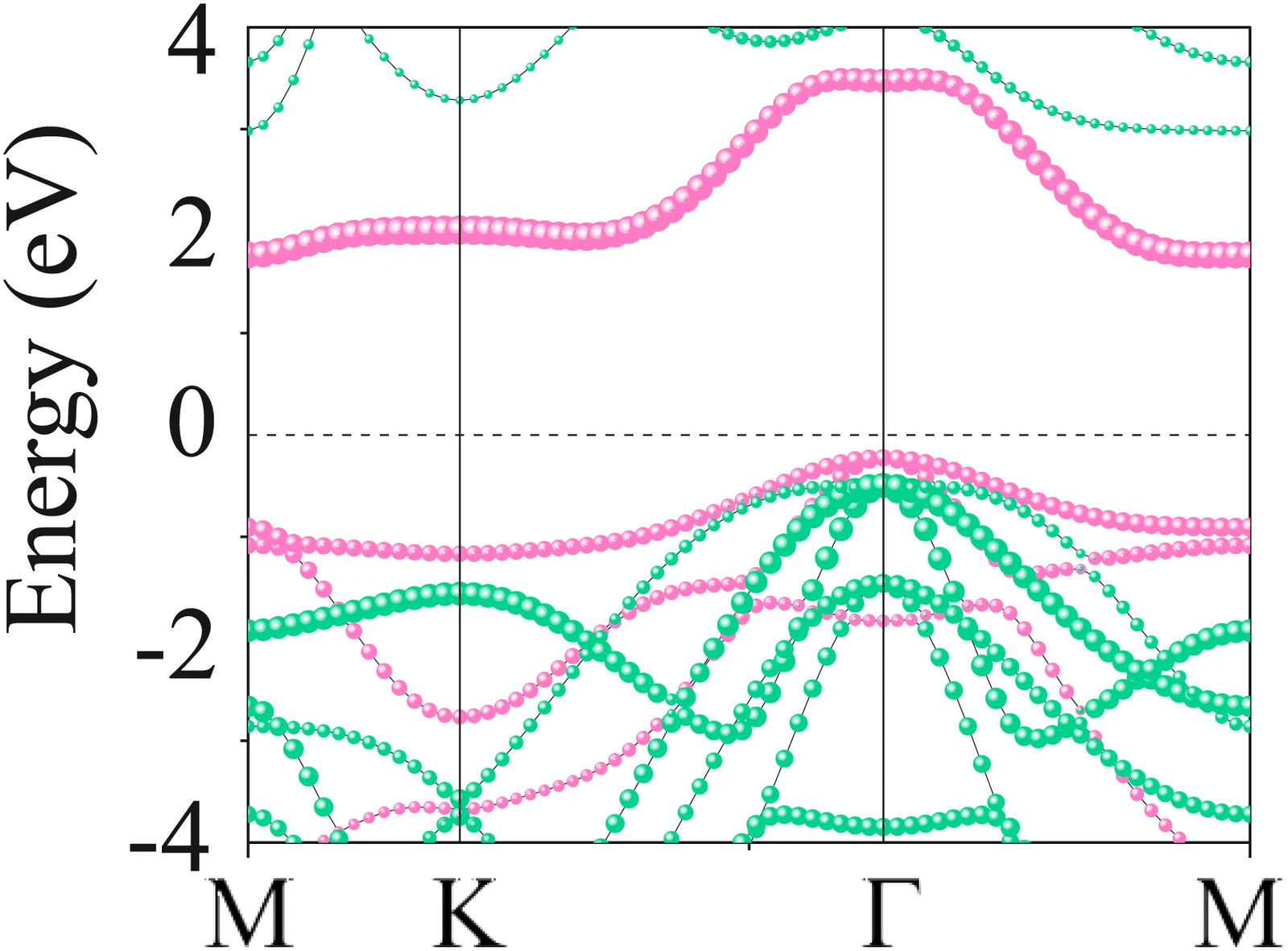
HSE06 projected band structure of the HfSO/BSe heterostructure under applied electric field of *E* = +0.1 V Å^−1^.

## 4 Conclusions

In summary, we have used first-principles calculations to characterize the structural, electronic, mechanical, and optical behavior of the BSe/HfSO vdW heterostructure. This heterostructure adopts a native staggered type-II band configuration with a gap substantially narrower than those of the isolated layers, while work-function and charge-density-difference analyses reveal spontaneous electron transfer from the BSe layer to the HfSO layer, leading to the formation of an intrinsic built-in electric field that may facilitate spatial separation of photoexcited carriers. Furthermore, the heterostructure exhibits significantly enhanced visible-light absorption and a competitive power conversion efficiency of 4.27%, highlighting its potential for ultrathin photovoltaic applications. Applying a perpendicular electric field provides continuous control over the band gap and can induce a transition from the staggered type-II state to a type-I configuration. Such electrostatic controllability provides a route for tuning the band gap and band-edge alignment, and consequently the electronic-structure characteristics of the heterostructure. These findings provide valuable theoretical guidance for the design and development of Janus-based two-dimensional heterostructures with controllable electronic and optical properties.

## Conflicts of interest

There are no conflicts to declare.

## Data Availability

The data that support the findings of this study are available from the corresponding author upon reasonable request.
